# Indigo naturalis‑associated ischemic injury of colorectal mucosa: A case series study

**DOI:** 10.3892/etm.2025.12818

**Published:** 2025-02-07

**Authors:** Yiheng Ke, Liang Xu, Qi Tang, Zheyu Ruan, Junjie Liu, Shuiliang Ruan

**Affiliations:** 1 Jiaxing University Master Degree Cultivation Base, Zhejiang Chinese Medical University, Hangzhou, Zhejiang 310000, P.R. China; 2 Department of Gastroenterology, The Second Affiliated Hospital of Jiaxing University, Jiaxing, Zhejiang 314000, P.R. China

**Keywords:** ischemic, indigo naturalis, colorectal mucosa, adverse effects, case series study

## Abstract

Indigo naturalis, a traditional Chinese herbal medicine characterized by its dark blue hue, is utilized in the treatment of a diverse array of diseases, including ulcerative colitis, psoriasis, oral ulcers, radiation proctitis, chronic myelocytic leukemia and herpes zoster. The common adverse effects associated with indigo naturalis include liver dysfunction, headaches, abdominal pain and nausea. Notably, case reports have documented instances of ischemic injury to the colorectal mucosa attributed to indigo naturalis. The present case series study aimed to elucidate the clinical characteristics of patients that experienced an ischemic injury of the colorectal mucosa due to consuming Chinese patent medicines containing indigo naturalis. The present study included 15 patients (mean age, 61.7±15.1 years; 5 male patients) that were admitted to the Second Affiliated Hospital of Jiaxing University (Jiaxing, China) between March 2013 and February 2016. The patients developed ischemic colonic mucosal injuries after consuming indigo naturalis through Chinese patent medicines, with an incidence rate of 1.3% (15/1,157 patients). Overall, 9/15 patients had idiopathic thrombocytopenic purpura and 6 patients had schistosomal liver fibrosis with hypersplenism but normal myeloproliferative thrombocytopenia. The duration of continuous treatment with indigo naturalis-containing Chinese patent medicines ranged from 12-330 days, with a mean of 98.0±52.3 days. Gastrointestinal symptoms, including abdominal pain, diarrhea and hematochezia, were observed 6-90 days after starting the indigo naturalis-containing Chinese patent medicines, with a mean onset of 28.9±22.2 days. Abdominal computed tomography scans and colonoscopy revealed lesions predominantly in the right hemi-colon and the entire colon, including the rectum. Gastrointestinal symptoms resolved 17.3±12.4 days after discontinuing the treatment with indigo naturalis-containing Chinese patent medicines, and patients had a favorable prognosis and did not experience a recurrence of ischemic colitis. Therefore, individuals taking indigo naturalis orally may be susceptible to developing ischemic injuries of the colorectal mucosa. In cases where colonoscopy indicates a suspected mucosal ischemic injury, it is suggested that endoscopists inquire about the medical history of the patient in order to establish a definitive diagnosis and adjust the dose of the indigo naturalis-containing drug to mitigate the adverse effects.

## Introduction

Ischemic colitis (IC) is characterized by ischemic changes in the intestinal mucosa due to non-occlusive damage to small blood vessels (1-3). There has been a gradual increase in the prevalence of IC in recent years. A population-based retrospective cohort study indicated that the incidence of IC increased by approximately four-fold, from 6.1 cases/100,000 individuals/year in 1976-1980 to 22.9 cases/100,000 individuals/year in 2005-2009(4). A 2024 Japanese study revealed that the incidence of ischemic colitis increased from 20.8 to 34.9 per 100,000, representing an ~1.6-fold increase (5). While IC is more common in older patients (>60 years) with underlying atherosclerosis, IC is also associated with certain medications, including psychotropic drugs (such as quetiapine and clozapine), digoxin, oral contraceptives, immunomodulators [such as lenalidomide, corticosteroids and tumor necrosis factor (TNF)-α inhibitors], laxatives and non-steroidal anti-inflammatory drugs (6). Thus, identifying drug-induced IC and implementing accurate management strategies is important for the effective and prompt control of the disease.

Evidence suggests that herbal medicines containing components of indigo naturalis may contribute to the pathogenesis of IC (7-10). Indigo naturalis is a dried powdered mass obtained from processing leaves or stems of plants such as *Baphicacanthus cusia* Bremek, *Polygonum tinctorium* Ait and *Isatis indigotica* Fort (11,12). This dark blue compound has been used in Traditional Chinese medicines for thousands of years for its therapeutic properties, and may be used to treat conditions such as hemoptysis, epistaxis and infantile convulsion as well as for the management of psoriasis and ulcerative colitis (UC). Indigo naturalis contains >63 identified biologically active compounds, including indole alkaloids, terpenoids, organic acids, steroids and nucleosides. Indirubin, an indole alkaloid, is recognized for its therapeutic potential, particularly in treating leukemia and as an anti-inflammatory agent. The chemical diversity of indigo naturalis contributes to its broad range of pharmacological activities, including its anti-inflammatory, antimicrobial and antitumor properties (10,13). Despite these therapeutic applications, there are concerns regarding the potential adverse effects associated with its long-term use, which highlights the need to investigate its safety profile (10,13). Various formulas of Traditional Chinese medicine contain indigo naturalis, such as indigo naturalis pills, powder and Sheng-Xue-Xiao-Ban capsules. Indigo naturalis in compound formula products, such as Sheng-Xue-Xiao-Ban capsules, is commonly used to treat immune thrombocytopenic purpura, psoriasis and UC (7,14-16). Additonally, Sheng-Xue-Xiao-Ban capsules are commonly used to treat hypersplenism-induced thrombocytopenia and to promote normal bone marrow cell proliferation in Jiaxing (Zhejiang, China), a region where schistosomiasis was prevalent with a number of middle-aged (45-60 years) and elderly patients (>60 years) still suffering from schistosomiasis hepatic fibrosis (17). As a result, indigo naturalis is widely prescribed to patients in this region.

Ischemic injury to the colorectal mucosa associated with indigo naturalis has a manifestation that is similar to transient IC, characterized by segmental distribution, mucosal hemorrhage, erosion and ulcer formation (7-9). From 2004 to June 2012, the China National Center for Adverse Drug Reaction Monitoring reported 344 cases associated with compound formulas such as indigo naturalis pills, capsules and tablets. These patients primarily had digestive system complaints, and presented with symptoms such as hepatitis, abnormal liver function, diarrhea, abdominal pain and dizziness. Among the reported cases, 23 were classified as severe due to drug-induced liver injury and gastrointestinal bleeding (1).

There has been a notable increase in IC morbidity to date (4,5). Previous studies have identified patients with atypical ischemic colorectal mucosal injury with a history of taking indigo naturalis orally (8,9). Furthermore, the clinical symptoms of these patients were alleviated when the medication was discontinued (8,9). Therefore, patients that exhibit ‘ischemic-like changes in the colorectal mucosa’ during colonoscopy should be investigated further, especially patients that have historically taken indigo naturalis medication. Therefore, the present study aimed to elucidate the clinical characteristics of patients that experienced ischemic injury to the colorectal mucosa when it was associated with the consumption of Chinese patent medicines containing indigo naturalis.

## Patients and methods

### Study design and population

The present case series study included patients that developed ischemic colonic mucosal injury after consuming Chinese patent medicines containing indigo naturalis between March 2013 and February 2016 at the Second Affiliated Hospital of Jiaxing University (Jiaxing, China). Patients were included if the following inclusion criteria were met: i) Gastrointestinal symptoms occured within 6 months of consuming Sheng-Xue-Xiao-Ban capsules containing indigo naturalis (oral consumption, 1.8 g per dose, three times a day); ii) ischemic injury of the colorectal mucosa was diagnosed according to clinical symptoms and colonoscopic findings based on the American College of Gastroenterology (ACG) clinical guidelines (18); and iii) mucosal recovery to normalcy was confirmed by another colonoscopy after drug discontinuation. Patients were excluded based on the following exclusion criteria: i) A previous medical history of IC; ii) the presence of other conditions, such as portal hypertensive colopathy, venous sclerosing enteropathy, hernia, intussusception, volvulus, intestinal obstruction, intestinal tumors or other diseases known to cause ischemic colonic mucosal injury; iii) conditions that could not be clinically distinguished from inflammatory bowel disease, infectious colitis or pseudomembranous enteritis; iv) the use of other medications known to cause IC within 6 months, including psychotropic drugs (such as quetiapine and clozapine), digoxin, oral contraceptives, immune-modulators (such as lenalidomide, corticosteroids and TNF-α inhibitors), laxatives and non-steroidal anti-inflammatory drugs; and v) patients with incomplete clinical data.

Diagnostic criteria for ischemic injury of the colorectal mucosa were based on ACG clinical guidelines and included: i) Clinical symptoms, such as abdominal pain, hemafecia or diarrhea; ii) colonoscopy findings that indicated intestinal wall ischemia or intraoperative colonic ischemia and necrosis; and iii) abdominal computed tomography (CT) findings of intestinal wall thickening, intestinal lumen stenosis, intestinal dilatation or pneumoperitoneum, with or without additional CT angiography or abdominal vascular ultrasound suggesting abdominal vascular stenosis or occlusion. The study protocol was approved by the Ethics Committee of The Second Affiliated Hospital of Jiaxing University (Jiaxing, China; approval no. 2023-ZFYJ-048-01). As the present study was retrospective, informed consent was waived.

### Data collection

Clinical data, including sex, age, date of initial medication, duration of uninterrupted medication, time between initial medication and onset of gastrointestinal symptoms, underlying medical conditions, past surgical history, presenting symptoms, abdominal CT scan results, colonoscopy findings, histological examination of intestinal mucosa, treatment modalities, prognosis, and follow-up outcomes, were collected retrospectively for the period from March 2013 to February 2016 through the medical record system and endoscopy system of The Second Affiliated Hospital of Jiaxing University.

### Staining method

The staining method utilized for histopathological examination was hematoxylin and eosin staining. Tissue specimens were subjected to fixation in a 10% neutral buffered formalin solution for a period ranging from 12 to 24 h, and maintained at ambient temperature. Following fixation, the tissues were processed for paraffin embedding and subsequently sectioned at a precise thickness of 4 µm. The staining protocol entailed the following steps: Initially, the sections were stained with hematoxylin solution for a duration of 5 to 10 min at room temperature; subsequently, they were stained with eosin solution for 2 to 3 min, under the same temperature conditions. The stained sections were then meticulously examined and analyzed utilizing a conventional optical microscope.

### Statistical analysis

The distribution of continuous variables was assessed for normality using the Shapiro-Wilk test, a widely accepted method for small to moderately-sized samples. Variables that passed the normality test (P>0.05) were considered normally distributed and reported as mean ± standard deviation (SD). Categorical data are presented as frequencies and percentages. Statistical tests were conducted using basic descriptive statistics suitable for the type and distribution of the data. P<0.05 was considered to indicate a statistically significant difference.

## Results

**Figure 1:**
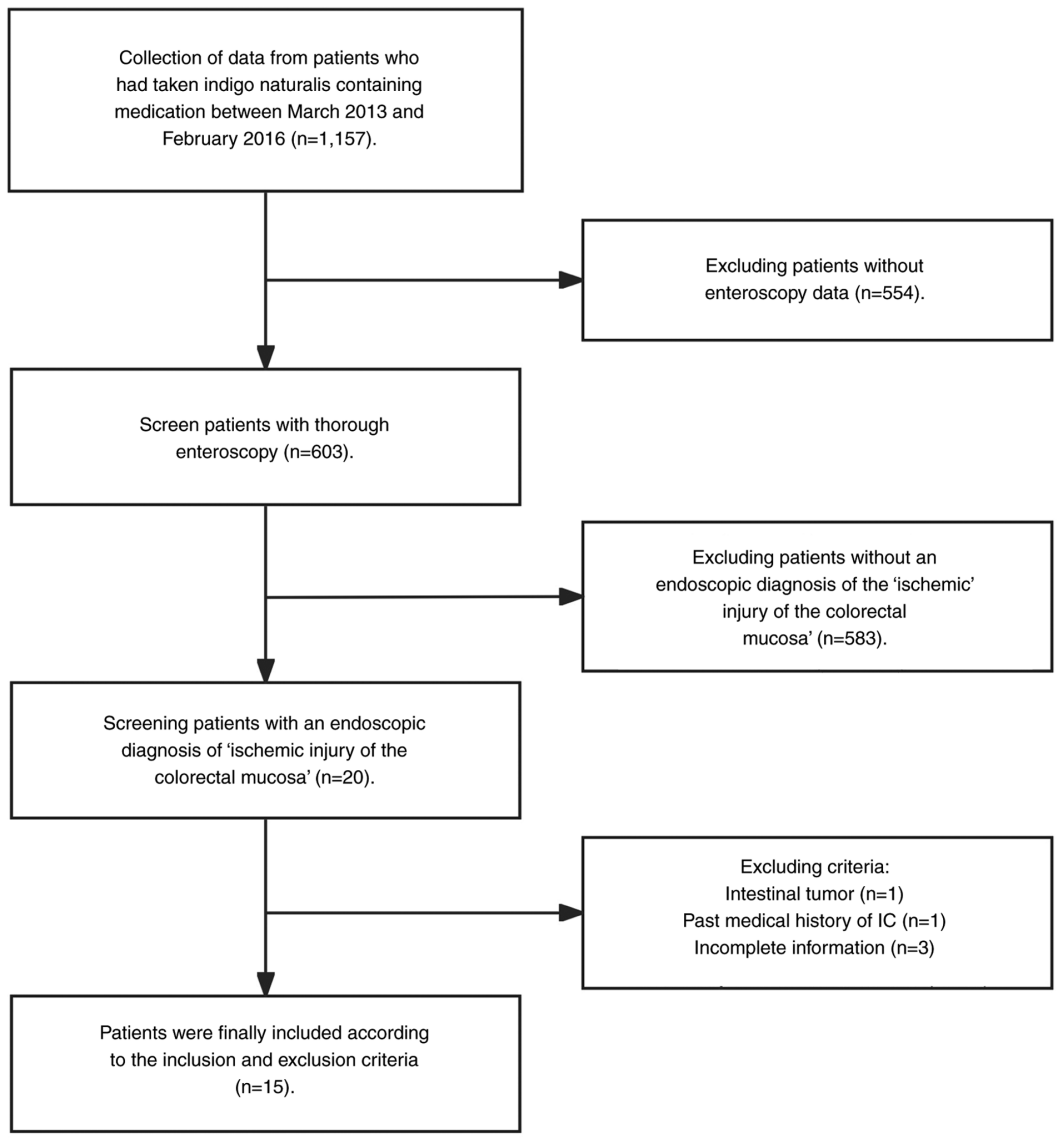
Flowchart of patient inclusion and exclusion process in this study.

A total of 1,157 patients had consumed indigo naturalis. However, 554 patients that lacked colonoscopy data, 583 patients without evidence of ischemic colorectal mucosal injury, 1 patient with intestinal tumors, 1 patient with a prior history of IC and 3 patients without complete data, were excluded. Therefore, 15 patients (mean age, 61.7±15.1 years; 5 males) with ischemic injury of the colorectal mucosa attributed to indigo naturalis consumption (Sheng-Xue-Xiao-Ban capsules containing indigo naturalis; 1.8 g per dose, three times a day) were included in the present study (Fig. 1).

Therefore, the overall incidence was 1.3%. Of the 15 patients included, 11 patients were admitted to an inpatient setting and 4 patients attended outpatient care. Abdominal pain or discomfort was reported by 14 patients (93.3%), diarrhea was reported by 9 patients (60.0%) and hemafecia was reported by 8 patients (53.3%). Additionally, 9 patients had idiopathic thrombocytopenic purpura and 6 patients indicated schistosomal liver fibrosis associated with hypersplenism and normal myeloproliferative thrombocytopenia. In addition, 5 patients had a history of hypertension and 4 patients had type 2 diabetes mellitus. The patients continuously consumed Sheng-Xue-Xiao-Ban capsules for 12-330 days, with a mean of 98.0±52.3 days. Symptom onset occurred between 6 and 90 days from initial drug therapy, with a mean of 28.9±22.2 days.

**Figure 2:**
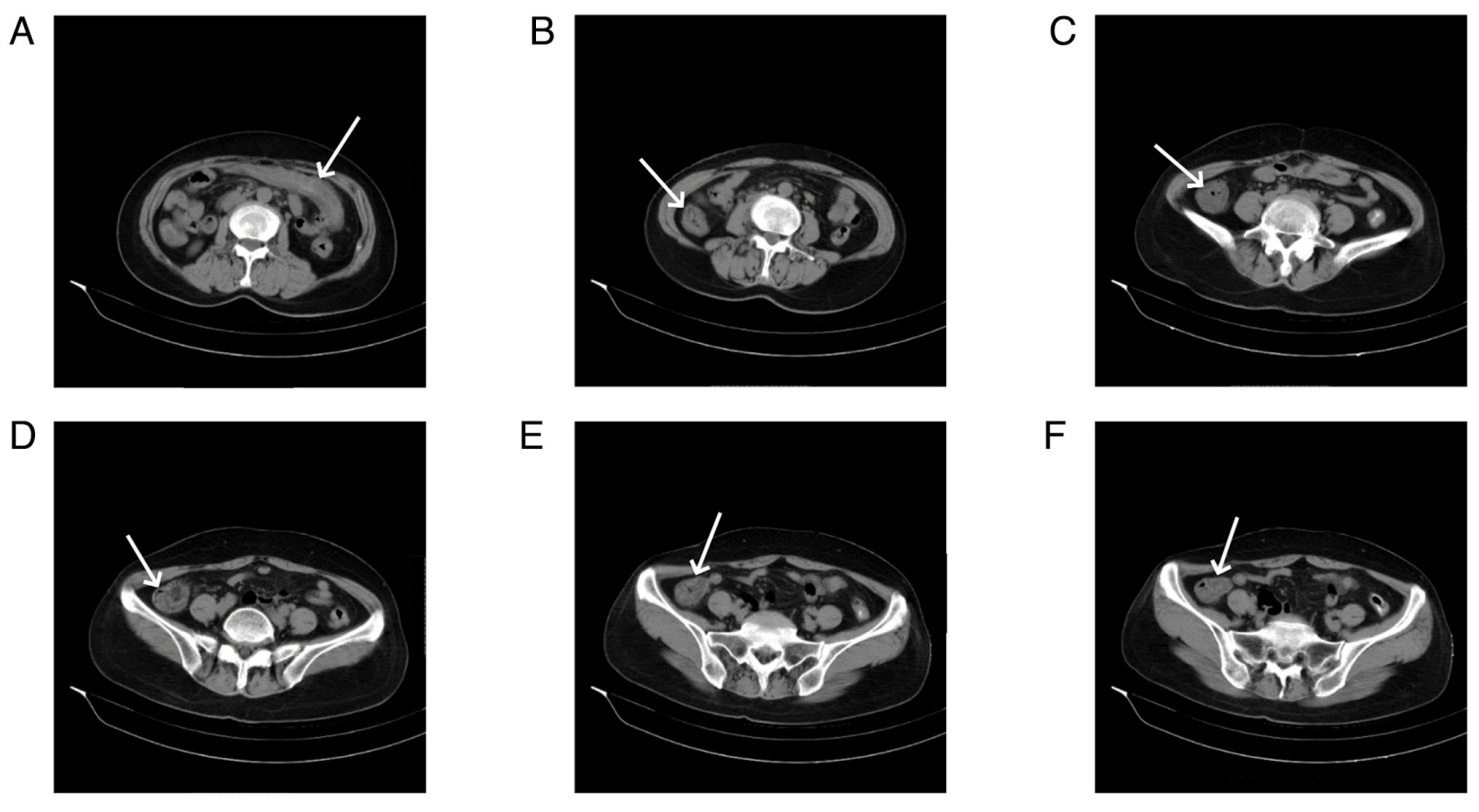
Abdominal computed tomography scan images of a patient with involvement of the right colon. (A) Edema and a thickened bowel wall (arrow) in the transverse colon was observed. A thickened bowel wall (arrows) was observed in the (B-D) ascending colon and (E and F) ileocecal region.

**Figure 3:**
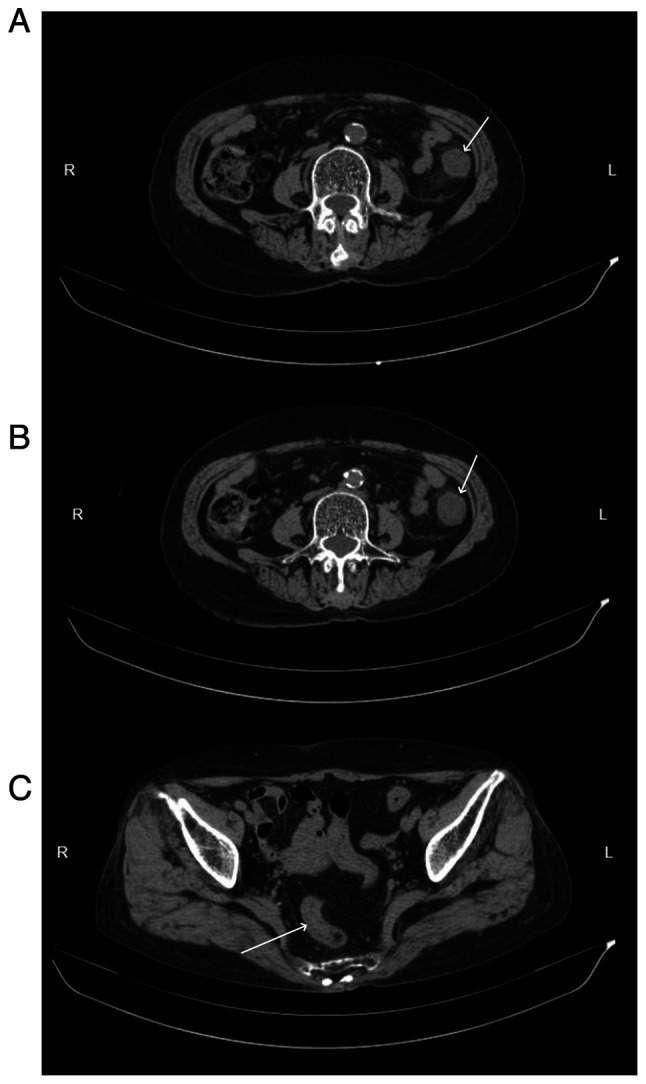
Abdominal computed tomography scan images of a patient with involvement of the left colon. (A and B) Edema and a thickened bowel wall (arrows) in the descending colon was observed. (C) A thickened bowel wall (arrow) was observed in the sigmoid colon. R, right side of the patient; L, left side of the patient.

**Figure 4:**
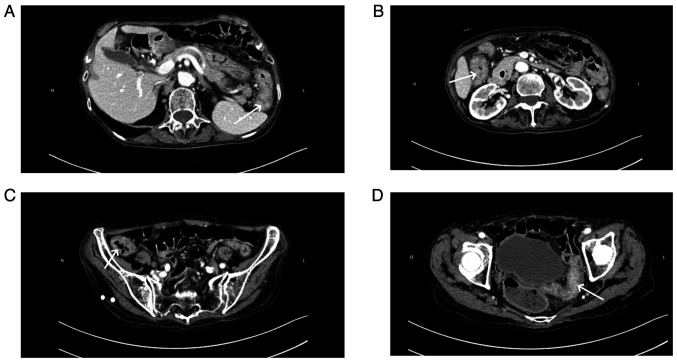
Abdominal contrast-enhanced computed tomography scan images of a patient with involvement of the total colon. (A) A thickened colonic wall (arrow) at the splenic flexure was observed. Edema and a thickened bowel wall (arrows) in the (B and C) ascending colon and (D) sigmoid colon were observed. R, right side of the patient; L, left side of the patient.

**Figure 5:**
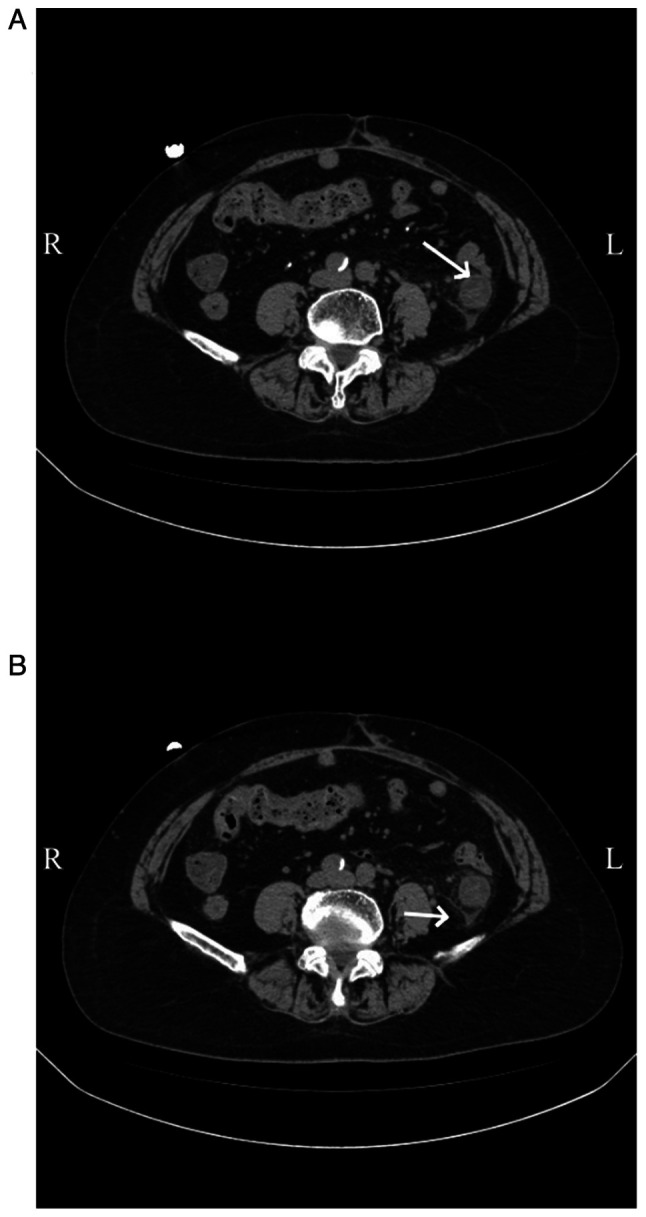
Abdominal computed tomography scan images of a patient with exudation around the descending colon accompanied by an abdominopelvic effusion. (A) Edema (arrow) in the descending colon with surrounding exudation. (B) Abdominopelvic effusion (arrow). R, right side of the patient; L, left side of the patient.

Abdominal CT scans, both plain and enhanced, were available for 11 patients, and had all been carried out within 1 week of the onset of abdominal symptoms. The right hemi-colon (Fig. 2) was affected in 4 patients (36.36%), the left hemi-colon (Fig. 3) in 2 patients (18.18%) and the total colon (Fig. 4) in 4 patients (36.36%). Additionally, 1 patient had periintestinal exudation (Fig. 5), while 4 patients had abdominopelvic effusion. The abdominal CT scan of 1 patient showed no abnormalities. Abdominal CT plain scans involving the transverse and ascending colon of a patient are presented in Fig. 2. CT images of a patient with the descending colon and sigmoid colon involved are presented in Fig. 3. Enhanced CT images of a patient with the total colon involved are presented in Fig. 4. CT images of a patient with exudation around the descending colon accompanied by an abdominopelvic effusion are presented in Fig. 5.

**Figure 6:**
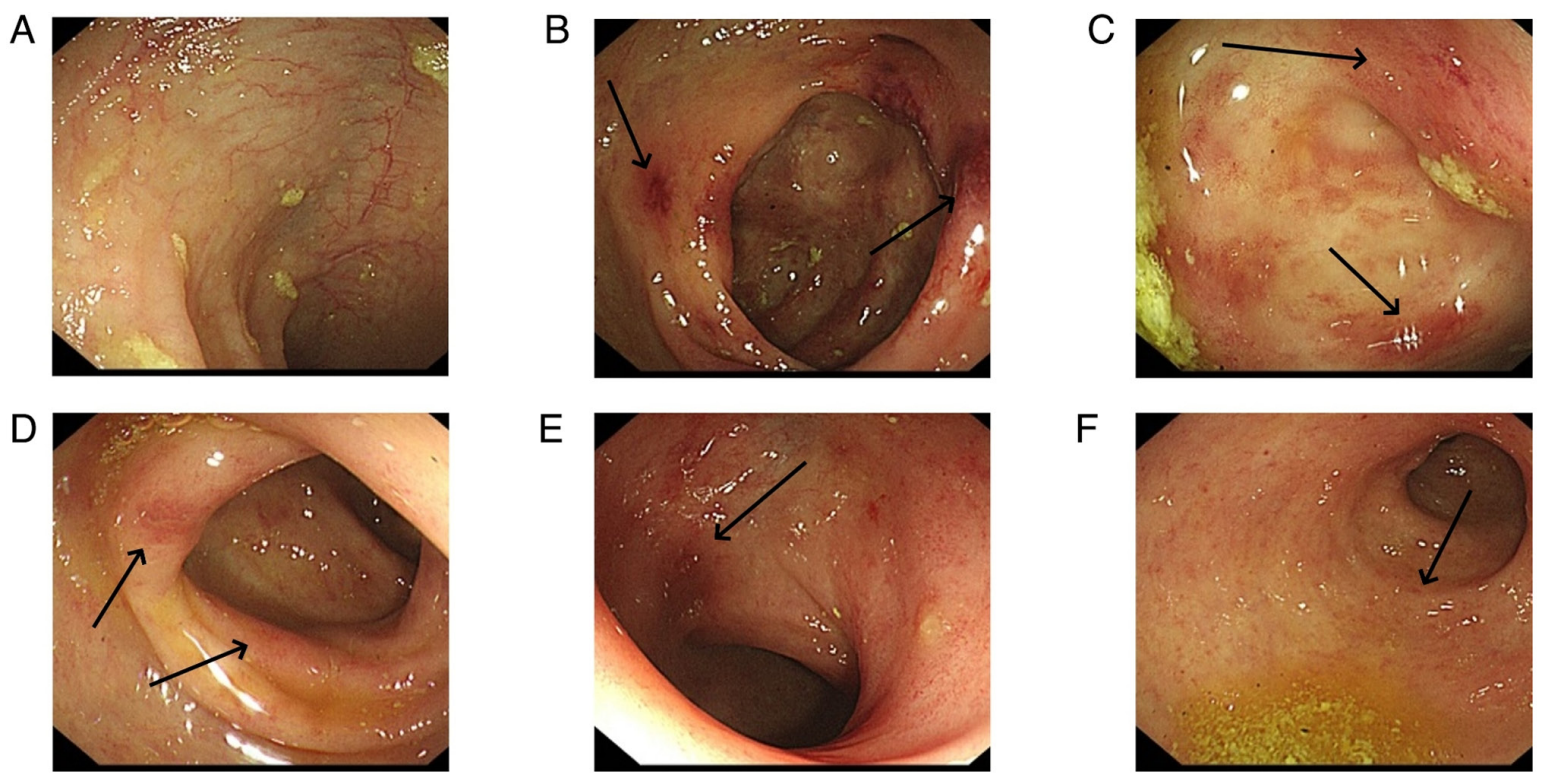
A colonoscopy of a patient with involvement of the total colonic mucosa. (A) An ischemic manifestation in the mucosa of the terminal ileum was not observed. Hemorrhagic lesions (arrows) were observed in the mucosa of the (B and C) ileocecal region, (D) descending colon and (E) sigmoid colon. (F) An area of rectal mucosal involvement (arrow).

**Figure 7:**
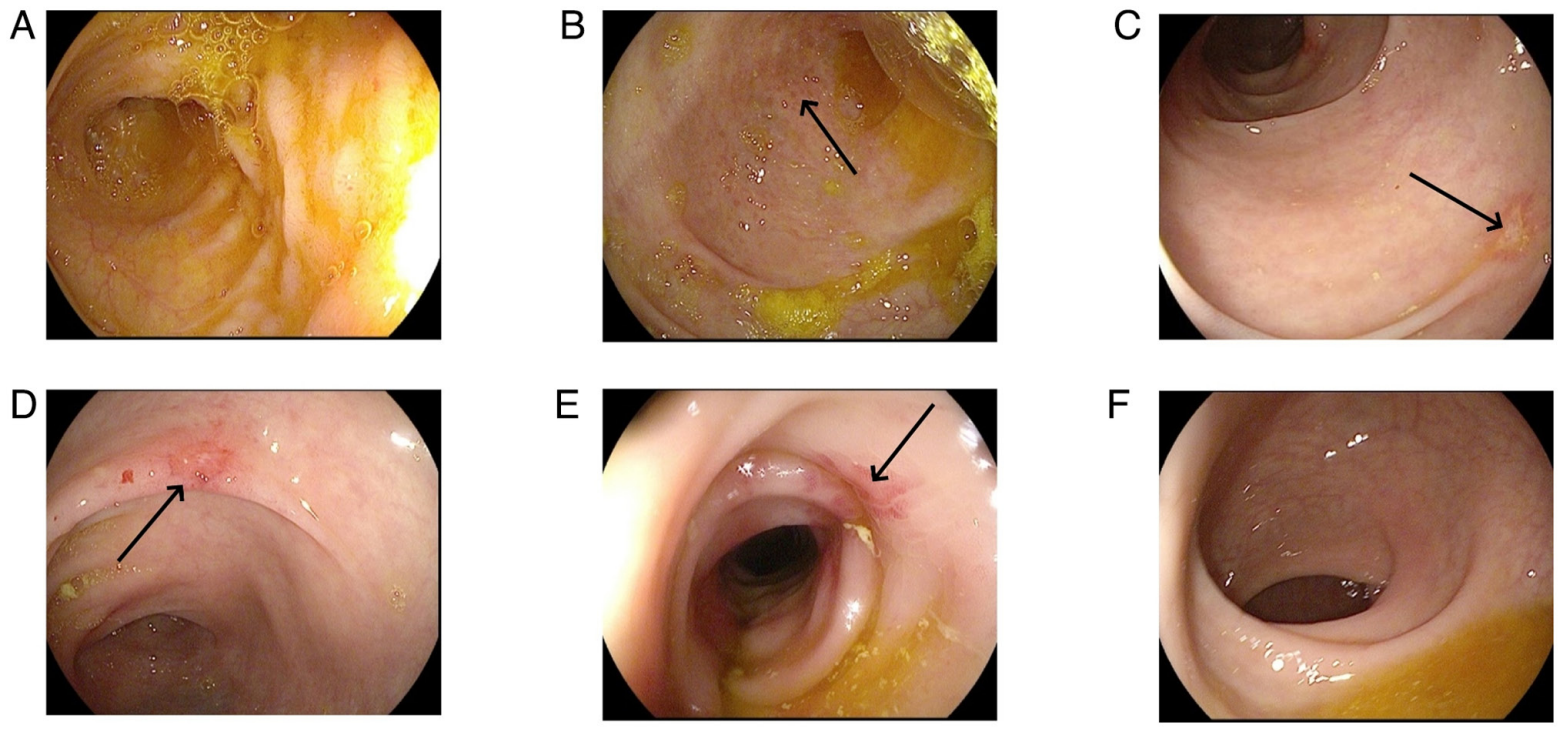
A colonoscopy of a patient with an ulcer as the main manifestation. (A) An ischemic manifestation in the mucosa of the terminal ileum was not observed. (B) Scattered, small, discoid-shaped foci of erosions (arrows) were observed in the ileum. (C and D) Scattered foci of ulceration (arrows) were observed in the descending colon. (E) Ischemic foci (arrow) were revealed in the sigmoid colon. (F) Ischemic foci were not observed in the rectum.

**Figure 8:**
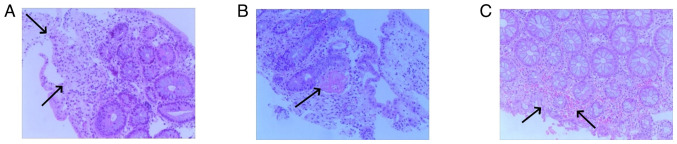
Typical pathological images of ischemic injury to the colorectal mucosa. Hematoxylin-eosin staining at x100 magnification. (A) Epithelial detachment and mucosal necrosis, with notable infiltration of inflammatory cells (arrow). (B) Intravascular thrombosis (arrow) was observed within the submucosal layer. (C) A shedding necrosis of the superficial layer of the mucosal epithelium was observed with an aggregation of erythrocytes (arrow).

Colonoscopy revealed that the total colon was affected in 9 patients (60.0%), the left hemi-colon in 5 patients (33.3%) and the right hemi-colon in 1 patient (6.7%). Rectal involvement was observed in 12 patients (80.0%). In 6 patients, the end of the ileum was reached during the colonoscopy, but it revealed that the small bowel was not involved. The typical presentation of the total colon and rectal involvement is presented in Fig. 6. Fig. 7 demonstrates 1 case that is primarily characterized by ulceration. Pathological examination was carried out for 7 patients and revealed mucosal superficial epithelial detachment and necrosis, fibrinoid exudation and inflammatory cell infiltration (Fig. 8). The clinical and pathological data for all 15 patients is presented in Table I.

**Figure 9:**
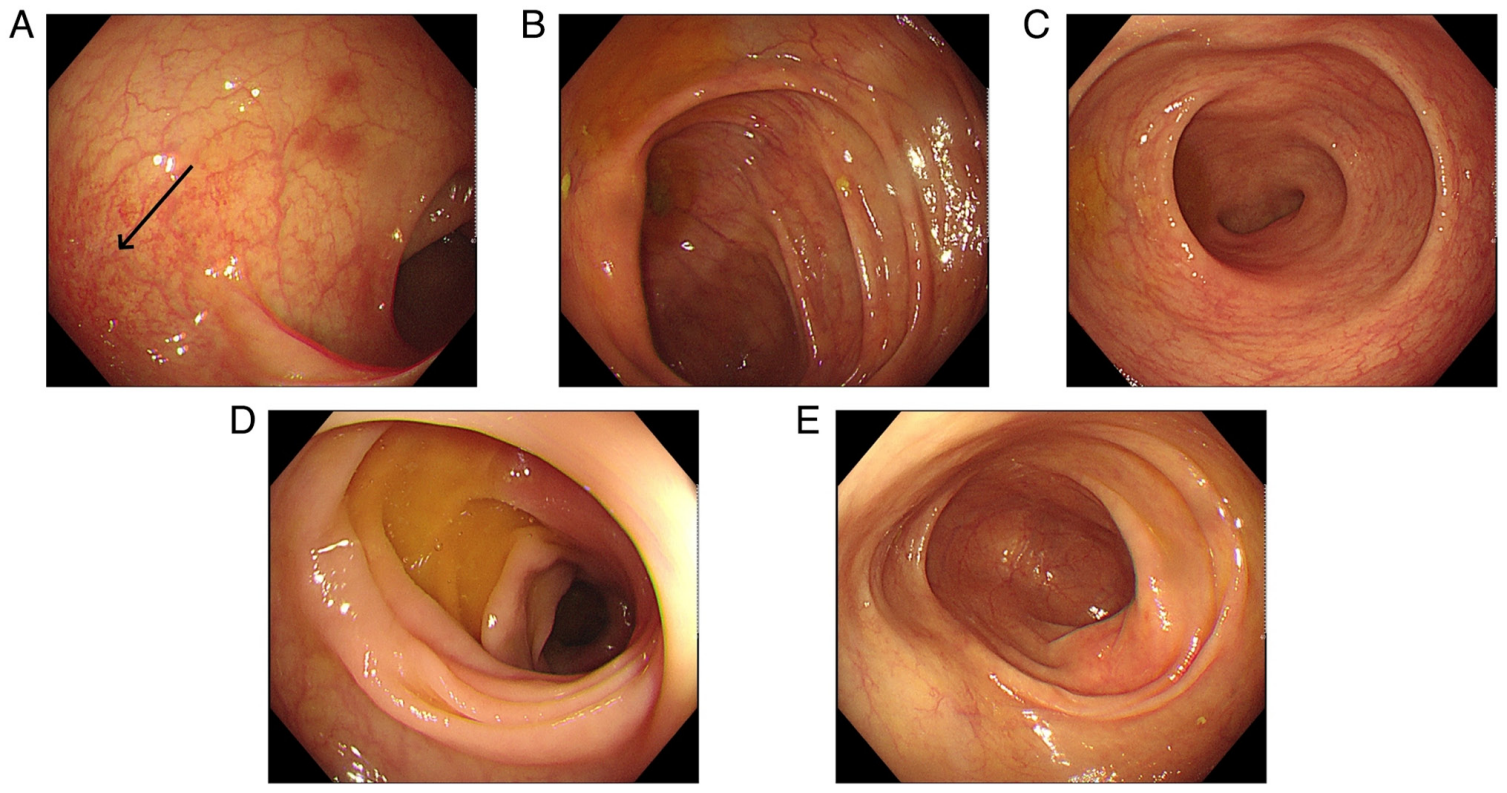
Recovery phase images of a patient. (A) Hyperemia and edema (arrow) were observed in the mucosa of the descending colon. Ischemic manifestations in the mucosa of the (B) ileocecal region, (C) rectum, (D) sigmoid colon and (E) transverse colon were not observed.

In 4 cases, gastroenterological symptoms and intestinal lesions resolved ~1 week after discontinuing Sheng-Xue-Xiao-Ban capsules without additional treatment. Due to symptoms such as abdominal pain, diarrhea and hemafecia, 6 patients were hospitalized in the Department of Gastroenterology of the Second Affiliated Hospital of Jiaxing University, but were discharged after their symptoms abated. A total of 3 patients were hospitalized in the Department of Hematology of the Second Affiliated Hospital of Jiaxing University due to thrombocytopenia. During their hospital stay, they developed gastrointestinal symptoms and were subsequently transferred to the Department of Gastroenterology, where they discontinued the Sheng-Xue-Xiao-Ban capsules, resulting in symptom improvement. Due to abdominal pain, diarrhea and mucus stools, 1 patient was readmitted to the Department of Gastroenterology of the Second Affiliated Hospital of Jiaxing University after discontinuation of Sheng-Xue-Xiao-Ban capsules; however, complete symptom resolution occurred 40 days after drug withdrawal. Another patient had been diagnosed with UC in the outpatient clinic of the Second Affiliated Hospital of Jiaxing University and had orally consumed indigo naturalis and mesalazine (0.5 g per dose, three times a day) for 1 year before discontinuing the drugs. Subsequently, symptoms disappeared 3 months after discontinuing the drug. The mean time from drug withdrawal to the resolution of symptoms was 17.3±12.4 days. The therapeutic measures for all inpatients were mainly symptomatic treatments such as fasting and fluid infusion. Following the resolution of symptoms, all patients underwent colonoscopy, which revealed recovery of the intestinal mucosa. Fig. 9 presents the recovery phase images of the same patient as presented in Fig. 6. These images indicated hyperemia and edema in the descending colon, while the mucosa of the remaining colonic regions had no notable abnormalities. To date, none of the patients have had surgical intervention for intestinal necrosis or perforation.

## Discussion

The present study reported ischemic injury to the intestinal mucosa, which was associated with the consumption of indigo naturalis. Abdominal CT scans and colonoscopy revealed that lesions were predominantly located in the right hemi-colon and the total colon, with rectal involvement in a number of cases. After discontinuing medication, gastrointestinal symptoms resolved, and patients generally exhibited a favorable prognosis with no recurrence. Therefore, it is suggested that clinicians assess for ischemic injury to the intestinal mucosa in patients consuming indigo naturalis.

A total of 53.3% of the patients that were enrolled in the present study were elderly individuals (>60 years) who presented with comorbidities such as hypertension and diabetes mellitus. Female patients had an increased prevalence, consistent with previously reported findings (7). This suggested that older age, underlying comorbidities and being female may increase an individuals susceptibility to indigo naturalis-associated ischemic lesions in the colorectal mucosa. Clinical examination of the patients in the present study did not reveal notable physical signs, and were predominantly manifested as pressure discomfort in the left lower abdominal region or the lower abdomen, occasionally accompanied by mild muscular tension. Vital signs revealed minimal alterations. The interval between drug initiation and symptom onset ranged from 6-90 days, which was shorter compared with that reported in a previous study (7). This discrepancy may be attributed to inter-individual variability in drug sensitivity and may be associated with the small sample size used in the present study. In the present study, symptoms of diarrhea manifested earlier, with an increased intensity and duration compared with previous research (7). This observation was likely associated with the prokinetic effect of indigo naturalis on intestinal peristalsis (19).

Abdominal CT, coupled with oral and intravenous contrast agents (such as iohexol and iopromide), can serve as a promising imaging modality for assessing IC (20). The patients in the present study had a CT presentation similar to patients with IC, with the main manifestation being bowel wall edema and thickening (21); however, the lesions in the present patients were more extensive and frequently involved the right side of the colon.

Previous studies have demonstrated the therapeutic efficacy of indigo naturalis in experimental rat models of UC (22,23). A previous study summarized the therapeutic benefits of indigo naturalis in patients with UC, suggesting its potential to promote mucosal healing (24). Numerous medical institutions have incorporated indigo naturalis into their treatment protocols of UC (14,25); however, there are associated adverse events (25,26). In a national survey conducted in Japan, 877 of 49,320 patients with UC (1.8%) received indigo naturalis treatment. Of these 877 patients, 91 pateints experienced adverse events (liver dysfunction, gastrointestinal symptoms, headache, pulmonary arterial hypertension and intussusception), including 21 patients who reported gastrointestinal symptoms (nausea and epigastralgia). Furthermore, 8 patients developed colitis that was unrelated to UC (26). Cho *et al* (8) reported the case of a middle-aged female patient with a 2-year history of UC, who developed IC after discontinuing mesalamine by themselves and taking indigo naturalis (daily dose, 2 g) for 3 months. However, the patient did not have any pre-existing risk factors for IC, such as hypertension, diabetes mellitus, cardiovascular disease or a history of abdominal surgery (8). Additionally, a study by Kondo *et al* (9) also reported 2 patients with UC who developed intestinal wall thickening and edema while receiving indigo naturalis treatment. Symptoms reported by both patients included abdominal pain and bloody diarrhea (9). Furthermore, evidence suggests that indigo naturalis can influence the immune system (27). The study by Gu *et al* (28) demonstrated that the therapeutic efficacy of indigo naturalis in UC likely involves the activation of systemic immunity. This immune activation modulates multiple biological processes, including nuclear transcription, protein phosphorylation, cytokine activity, reactive oxygen species metabolism, epithelial cell proliferation and apoptosis. These effects are mediated through diverse mechanisms, such as Th17 cell differentiation, the Jak-STAT and IL-17 signaling pathways, and Toll-like and NOD-like receptors, as well as other cellular and innate immune signaling pathways. Nevertheless, further in-depth experimental studies are required to fully elucidate the underlying mechanisms. A number of studies also hypothesize that indigo naturalis can exacerbate colitis by disturbing the normal composition of the intestinal flora (29-33). Additionally, it has been hypothesized that indigo naturalis can aggravate IC through several mechanisms: i) Inducing diarrhea in susceptible individuals, which leads to a decreased blood volume and an increased intraluminal pressure and vascular spasms. This compromises the blood supply to the intestinal wall and results in degenerative and necrotic changes to the mucosal tissue with inflammatory cell infiltration. ii) The irritant affects the colonic mucosa, which causes damage to the intestinal mucosal lining. iii) Indigo naturalis has multi-target biological regulatory process effects, involving mechanisms such as anti-inflammatory action, immune modulation, antioxidant activity and stopping bleeding, and it exhibits a significant procoagulant effect. This procoagulant activity can lead to the formation of fibrin thrombi within blood vessels, resulting in vascular occlusion and subsequent ischemic necrosis of the colonic mucosa (19). Based on the findings of the present study, we hypothesize that indigo naturalis potentially has a key role in the development of ischemic injury and ulcer formation within the colorectal mucosa, although the precise underlying mechanism remains unclear.

Ischemic injury to the colorectal mucosa from indigo naturalis consumption closely resembles transient IC, which is characterized by segmental mucosal hemorrhage, erosion and ulceration (34). Manifestations include shallow ulcers similar to those in UC (35). However, UC typically presents as a continuous lesion, distinct from ischemic injury, which is frequently extensive and predominantly affects the entire colon, especially the right side and rectum (36). The diagnostic challenges arise due to the lack of awareness among endoscopists of the potential colorectal mucosal damage caused by indigo naturalis, which is attributable to the lack of access to the medication histories of patients. Consequently, the diagnosis frequently defaults to IC or UC. Endoscopists should proactively inquire about the medication histories of the patient when encountering atypical ischemic alterations in the colorectal mucosa. Pathological examination can reveal abnormalities such as superficial epithelial detachment, mucosal necrosis, fibrinoid exudation, hemosiderin granule deposition and submucosal vascular thrombosis, which aid in diagnosing ischemic lesions but may not indicate the specific etiology (7,19,37). Due to the retrospective nature of the present study and the unavailability of multiple colonoscopic reviews for all cases, precise data regarding the timing of colonic mucosal repair were not obtained.

The present study has several limitations. Firstly, it was conducted at a single center. Future research would benefit from a multicenter approach for a more comprehensive perspective. Secondly, all patients in the present study received the same dose of indigo naturalis (Sheng-Xue-Xiao-Ban capsules containing indigo naturalis, 1.8 g per dose, three times a day). Analyzing patients treated with varying doses could provide more robust data. Thirdly, expanding the cohort population would yield further valuable insights into the safety of the clinical application of indigo naturalis.

In conclusion, indigo naturalis may be associated with a notable incidence of ischemic injury to the colorectal mucosa. Any suspected mucosal ischemic injury during a colonoscopy should serve as a warning sign to proactively inquire about the medication history of the patient in order to make a precise diagnosis. Additionally, such injuries may resolve following drug discontinuation and may have a favorable prognosis.

## Figures and Tables

**Table I:** Clinical and pathological data of the patients.

Patient no.	Age, years	Sex	Section of colon affected	Pathological features revealed
1	27	Female	Sigmoid colon and descending colon	The superficial epithelium of the mucosa had shed and was necrotic, with an aggregation of red blood cells
2	60	Female	Rectum and total colon	Pathology not assessed
3	67	Male	Rectum, sigmoid colon and descending colon	Pathology not assessed
4	76	Female	Rectum and total colon	Pathology not assessed
5	52	Male	Rectum and total colon	The superficial epithelium of the mucosa had shed, with an infiltration of red blood cells
6	78	Female	Ascending colon and hepatic flexure	Pathology not assessed
7	65	Female	Rectum and total colon	Intestinal mucosal bleeding, epithelial shedding, mucus secretion and neutrophil exudation
8	75	Female	Total colon	Pathology not assessed
9	58	Female	Rectum and total colon	Thrombus formation within the submucosal blood vessels
10	55	Male	Rectum, sigmoid colon and descending colon	Chronic mucosal inflammation with vascular congestion and bleeding, and submucosal edema
11	64	Female	Rectum and sigmoid colon	The superficial epithelium of the mucosa had shed and was necrotic, with an infiltration of inflammatory cells
12	81	Female	Rectum and total colon	Pathology not assessed
13	35	Male	Rectum, sigmoid colon and descending colon	Pathology not assessed
14	73	Female	Rectum and total colon	The superficial epithelium of the mucosa had shed and was necrotic, with an infiltration of inflammatory cells and glandular atrophy
15	59	Male	Rectum and total colon	Pathology not assessed

## Data Availability

The data generated in the present study may be requested from the corresponding author.
